# Supplementary material for: Paclitaxel Induces the Apoptosis of Prostate Cancer Cells via ROS-Mediated HIF-1α Expression
Source: Molecules. 2022 Oct 24;27(21):7183. doi: 10.3390/molecules27217183 (PMC9654100; doi:10.3390/molecules27217183)
Supplement: Supplementary file 1 [file molecules-27-07183-s001.zip › molecules-1894991-supplementary.pdf]

molecules

Supplementary Information

Paclitaxel Induces the Apoptosis of Prostate Cancer Cells via ROS-Mediated HIF-1α Expression

Yan Zhang [1,†], Yedong Tang [2,†], Xiaoqiong Tang [1], Yuhua Wang [1], Zhenghong Zhang [2,*] and Hongqin Yang [1,*]

1 Key Laboratory of Optoelectronic Science and Technology for Medicine of Ministry of Education, Fujian Provincial Key Laboratory for Photonics Technology, Fujian Normal University, Fuzhou 350007, China
2 Fujian Provincial Key Laboratory for Developmental Biology and Neurosciences, College of Life Sciences, Fujian Normal University, Fuzhou 350007, China
* Correspondence: zhangzh@fjnu.edu.cn (Z.Z.); hqyang@fjnu.edu.cn (H.Y.)
† These authors contributed equally to this work.

Supplementary Figure S1

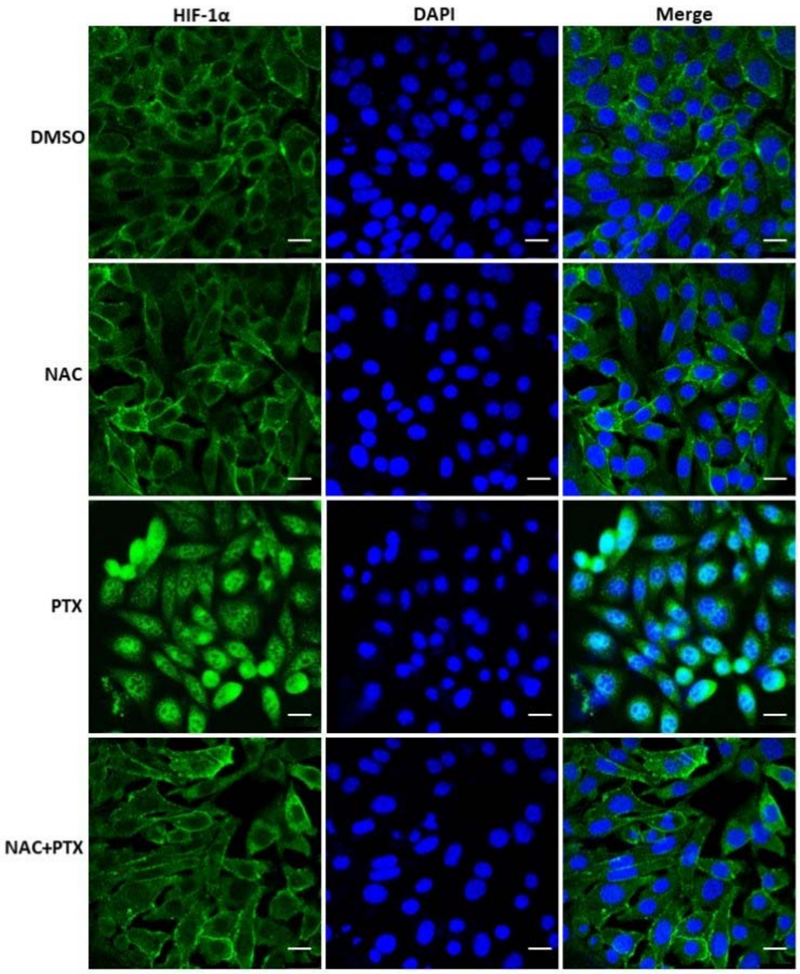

Figure S1. Nuclear localization of HIF-1α with treatment and with NAC in PC3M cells was examined using fluorescence microscope.

Supplementary Figure S2

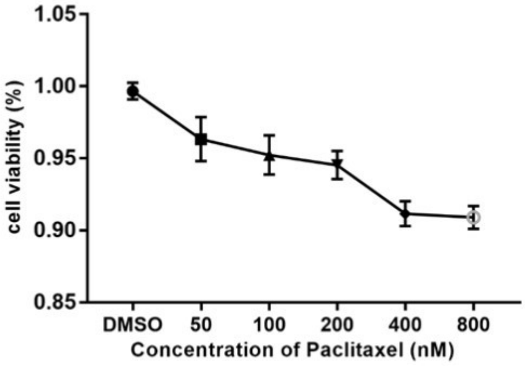

Figure S2. Cell viability of PC3M cells after treating with different concentrations of nM scale of paclitaxel.

Supplementary Figure S3

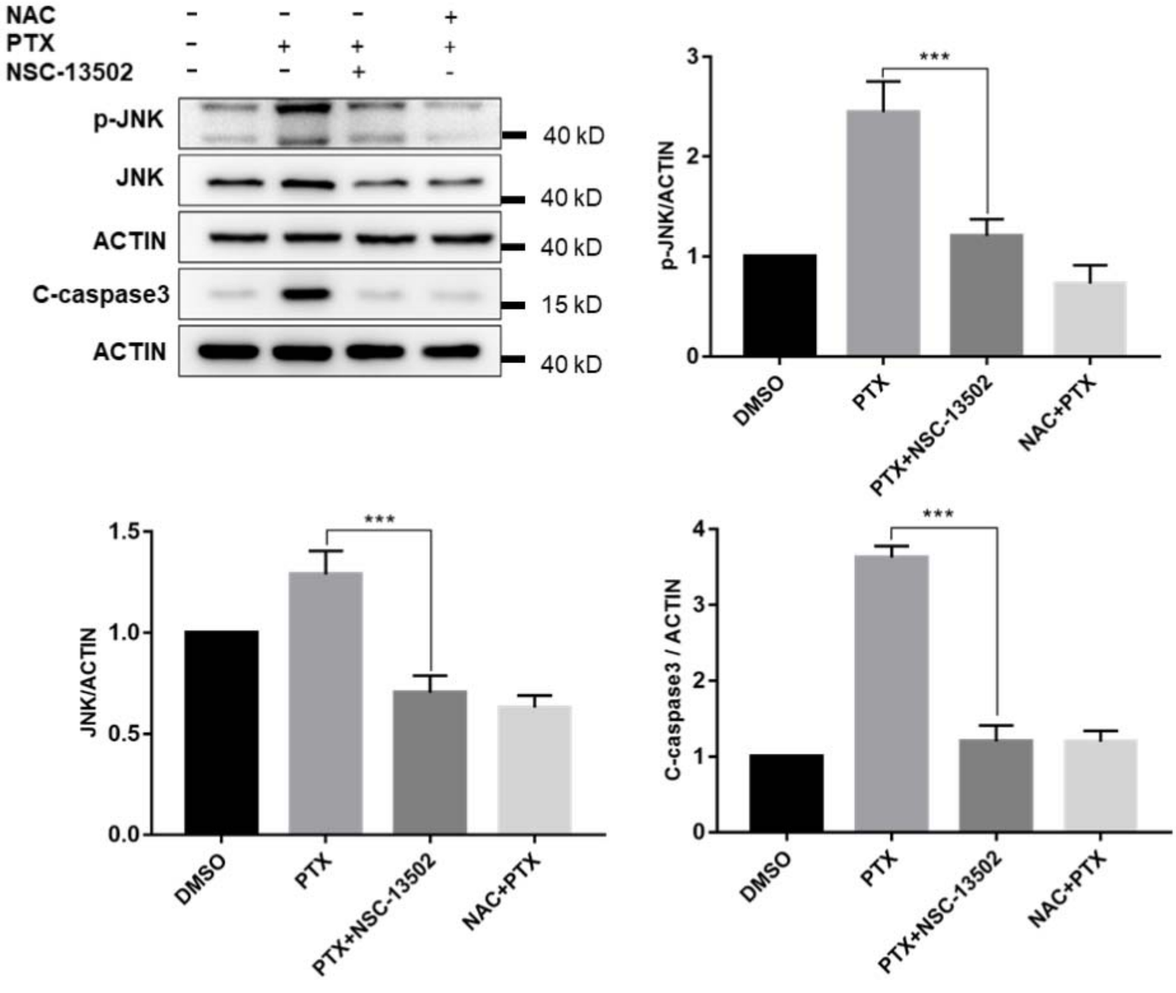

Figure S3. The expression of P-JNK, JNK, cleaved-caspase3 and actin in the PC3M cells were detected following the treatment with paclitaxel with or without NSC-13502. ***: $p < 0.001$.
